# Home-based exercise training in boys with Duchenne muscular dystrophy: A 12-month intervention study

**DOI:** 10.1177/18758894261446029

**Published:** 2026-05-11

**Authors:** Stian Hammer, Maria Vollsæter, Michel Toussaint, Ola Drange Røksund, Guri Brekke, Bjørge Herman Hansen, Haakon Krisitian Kvidaland, Lars Peder Bovim, Roy Miodini Nilsen, Tiina Andersen

**Affiliations:** 1 Department of Physiotherapy, 60498 Haukeland University Hospital, Bergen, Norway; 2 Department of Health and Functioning, 1657 Western Norway University of Applied Sciences, Bergen, Norway; 3 Department of Paediatric and Adolescent Medicine, Haukeland University Hospital, Bergen, Norway; 4 Department of Clinical Science, University of Bergen, Bergen, Norway; 5 Department of Neurology, Centre de Référence Neuromusculaire, ULB Hôpital Universitaire de Bruxelles (H.U.B.), Brussels, Belgium; 6 Department of Sport Sciences and Physical Education, 4343 University of Agder, Kristiansand, Norway; 7 Thoracic Department, Haukeland University Hospital, Bergen, Norway

**Keywords:** Duchenne muscular dystrophy, exercise training, physical activity, physical therapy, paediatric & adolescent rehabilitation

## Abstract

**Purpose:**

Duchenne muscular dystrophy (DMD) is a progressive X-linked neuromuscular disorder that reduces physical activity (PA) levels and leads to early loss of ambulation. The World Health Organization recommends promoting daily PA for children with disabilities. The primary aim of this study was to assess changes in PA level, physical function, and quality of life (QoL) after one year of a home-based exercise program in boys with DMD. Secondarily, it aimed to evaluate the suitability and safety of the exercise program for this patient group.

**Methods:**

In this prospective longitudinal intervention study, 12 boys with DMD (ambulatory and non-ambulatory) participated in an individually tailored program combining strength and endurance training three times per week for one year. Another 11 boys with DMD served as a non-intervention comparison group. PA-levels were monitored using accelerometery. Secondary outcomes included upper-limb function, lung function, grip strength, endurance, and health-related QoL.

**Results:**

Light and moderate-to-vigorous PA-levels increased in the intervention group but declined in the non-intervention group. Sedentary time increased in both groups. No adverse events were related to the intervention. While upper-limb function declined, other measures remained stable, with a small improvement in QoL.

**Conclusion:**

Home-based, moderate-intensity exercise appears to be beneficial and safe, and may sustain PA-levels, functional abilities, and QoL in boys with DMD.

## Introduction

Maintaining an active lifestyle has well-documented physical, psychological, and social benefits.^1,2^ Even modest increases in physical activity (PA) lead to improvements in cardiovascular fitness, muscular strength, mental health, and overall function in children with disabilities.^1^ To promote health and inclusion, the World Health Organization (WHO) recommends that children and adolescents, including those living with disabilities, engage in at least 60 min of moderate to vigorous PA (MVPA) daily, and perform muscle- and bone-strengthening activities at least three times per week.^1,3^

Duchenne muscular dystrophy (DMD) is a progressive X-linked neuromuscular disorder characterized by early motor delay, loss of ambulation typically in the second decade, and reduced life expectancy.^4^ Corticosteroid therapy can delay strength loss, improve cardiorespiratory function and prolong mobility,^5,6^ but disease progression remains inevitable.^4^ A recent study found that only 39% of ambulatory boys with DMD under the age of 12 met the WHO PA recommendations, compared to 71% of healthy controls. Activity levels declined with age in both boys with DMD and healthy controls, whereas activity patterns varied across functional stages.^7^

Evidence regarding structured, long-term PA interventions remains limited in DMD, particularly for those that combine both strength and endurance components.^8^ While PA is generally encouraged in DMD,^4,9^ there is a need for beneficial and safe exercise strategies that promote long-term engagement without accelerating disease progression.

This study aimed to evaluate potential benefits of a one-year, home-based, individually tailored strength and endurance training program on PA-levels, physical function, and quality of life (QoL) in boys with DMD. Secondarily, it aimed to evaluate the suitability and safety of the exercise program for this patient group.

## Methods

### Participants and study setting

Boys aged 6–18 years with a genetically and clinically confirmed diagnosis of DMD were recruited from Haukeland University Hospital's (HUH) children and youth clinic for an intervention group. They were eligible if they had no language or cognitive impairments that could interfere with completing questionnaires, following instructions, or participating in clinical and exercise tests, including accelerometer use. Boys with DMD aged 6–18 were also invited from children and youth clinics in the southeastern parts of Norway, meeting the same eligibility criteria, to participate in a non-intervention group for comparisons.

The study was approved by the Western Norway Regional Committee of Medical Research Ethics (2019/00260), and informed written consents were obtained from all participants prior to study start. This study was registered in ClinicalTrials.gov (NCT03963453) prior to the study start.

### Study design

A prospective longitudinal intervention study was conducted. After a four-week baseline registration for both groups, a 12-month intervention period with exercise training followed. The participants in the intervention group attended a five-days DMD camp at the study start, after six months, and at 12 months. Each five-day DMD camp included protocol-based multidisciplinary DMD follow-up, physical testing, and individual tailoring of the exercise training program. The camps were carried out at the HUH`s Vitality Center for children and youth,^10^ and the exercise training program was carried out as a home-based training program between the DMD camps (see Online Supplement 1). The non-intervention group received standard DMD care and participated in their regular daily activities without interventions.

### Baseline characteristics

Self-reported disease stages (e.g., early, late ambulatory and non-ambulatory stages based on the Vignos and Brooke Scales^11^), current treatments, and participation in PA were obtained from a questionnaire. The original Dutch questionnaire was developed by Heutinck et al.^12^ and translated into Norwegian, in collaboration with user representatives. Finally, it was digitalized using Checkware 3.2.1 software (Check Ware AS, Trondheim, Norway).

### Intervention

At the first DMD camp (baseline), an individualized exercise training program was developed for each participant by two experienced physiotherapists under the supervision of the lead clinical exercise physiologist. The program included a set of predefined exercises that were individually adapted to match each participant's functional abilities (see Online Supplement 2–3). To integrate the exercise program into daily routines, key support personnel were identified for each participant, including parents, school and personal assistants, and their local community physiotherapist. These key support personnel were trained to assist with the program and were informed about the exercise content, as well as when (day and time), where (at home, school, or their local physiotherapy clinic), and with whom the exercises should be performed. The exercise intervention consisted of both strength and endurance training, with two strength sessions and one endurance session per week. A minimum of 48 h of rest was ensured between the strength sessions. Longer periods of rest were encouraged, and adjustments were made as needed based on ongoing evaluation. A nutrition plan was not part of the intervention; however, participants were advised to consume a protein-rich meal shortly before and after each training session, in addition to general information about the importance of sufficient sleep routines.

The first three months of the intervention served as an adaptation period aimed at establishing safe and consistent exercise routines. It allowed participants, with assistance from their key support personnel, to gradually transition from an inactive to a more active lifestyle, develop proper training techniques, and become familiar with the tools used to regulate the exercise intensity (see Strength training). From month four onward, the focus shifted to ensuring progressive loading. Investigators provided a week-by-week digital training schedule created in Microsoft Excel 2021 (Microsoft Corporation, Redford, WA, USA), with exercise intensities adjusted as needed. Training sessions were recorded in a training diary by participants or their key support personnel. Each exercise was accompanied by illustrations, videos, and detailed instructions developed using Exorlive software (Exorlive, Oslo, Norway) (see Online Supplement 2–3). At the second DMD camp (six months after baseline), the feasibility of the home-based exercise program was evaluated. Adjustments were made if needed based on feedback from participants and key support personnel involved in sessions. The revised program was then followed until the 12-month follow-up.

#### Strength training

The strength training aimed to maintain or improve muscular strength and function, with emphasis on dynamic movements. Each program included six to eight exercises targeting major muscles and muscle groups in the lower limbs, upper limbs, back, and abdomen (see Online Supplement 2 and 3), and was adapted to both ambulatory and non-ambulatory participants using unilateral or bilateral movements. Resistance was provided through body weight, limb weight, adapted chair (pushing chair in ambulatory participants), weighted barrels or cuffs (0.5–2 kg loads), or different elastic bands. Active-assisted movement was allowed when needed (see Online Supplement 4). Exercise prescription was guided by the OMNI Resistance Exercise Scale and Repetitions in Reserve (RIR) to regulate resistance, sets and pauses.^13,14^ OMNI (0–10) enables self-assessed intensity, useful when maximal testing is inappropriate,^14^ while RIR estimates how many repetitions remain before failure, allowing daily adjustments based on daily physical readiness.^13^ Participants and their support personnel were trained in both tools. In the first three months (adaptation phase), a number of repetitions and sets of each exercise were performed to target OMNI 4 and RIR 4–6 (light intensity). From months four to six, repetitions and sets were targeted at OMNI 4–5 and RIR 3–5, and in the final six months, exercises progressed to moderate intensity at OMNI 5–6 / RIR 3–4. Rest intervals were three minutes for leg exercises, 30 s for core, and 1–2 min for upper extremities. Training diaries were reviewed weekly, and adjustments were made by the investigators. In some cases, additional exercises were gradually added.

#### Endurance training

The endurance training aimed to maintain or improve cardiorespiratory fitness. Activity types were selected during a dialogue with participants and their caregivers, based on individual preferences and access to equipment (e.g., THERA-trainer Tigo 510 [Medica Medizintechnik GmbH, Hockdorf, Germany] and MOTOmed [RECK-Tecknik GmbH & Co, Betzenweiler, Germany], motor-assisted arm/leg cycling devices; Innowalk [Made for Movement, Skien, Norway], a robotic-assisted standing and walking device), virtual reality games and pools (see Online Supplement 4). To promote variation, motivation, and feasibility, participants were offered multiple activity options. During the DMD camps, both activity types and training methods (e.g., intervals vs. continuous sessions) were tested, discussed, and adjusted as needed. In the first three months, sessions lasted at least 15 min. During months four to six, the duration gradually increased to 30 min and remained stable thereafter. The Borg Rating of Perceived Exertion (6–20 scale) was used to guide exercise intensity, aiming for a level of 11–13 (“somewhat hard”), where 6 represents no exertion and 20 maximal exertion.^15^ Endurance training was recorded in the participants’ exercise diaries and adjusted weekly by investigators using the same procedure as strength training.

### Exercise precautions

Both the participants and key support personnel were also instructed in the use of the Visual Analogue Scale (VAS) to document any pain experienced during or after training sessions,^16^ along with comments in the exercise diary. They were additionally asked to report prolonged post-session fatigue (>4 h). Reports of either sustained pain or reduced motivation triggered follow-up, and if needed, home visits to adjust the program and support adherence. During each five-day DMD camp, blood samples were collected at days one and five to monitor the participants’ serum creatine kinase (CK) levels as a proxy for potential muscle damage resulting from the total load of the exercise training program and activities of daily living.^17,18^

### Primary outcome

The intensities of PA were measured using a previously described setup with ActiGraph GT3X + accelerometers (ActiGraph, Pensacola, FL, USA) in the intervention group and the non-intervention group.^7^ The participants’ movements were recorded in three axes, providing an objective measure of the intensity, frequency, and duration of the activity performed during wear time, summarized as count units. The data were processed using ActiLife v.6.13.3 software (ActiGraph, Pensacola, FL, USA) . Average counts per minute (CPM) were calculated by dividing the total number of counts by the total number of valid wear minutes. Intensity-specific PA-levels were determined by applying commonly used cut-points (see below).

The non-ambulatory boys used one accelerometer on the wrist of their dominant hand, whereas ambulatory boys with DMD used two accelerometers: one on their right hip and one at the wrist of the dominant hand. Participants were instructed to wear the accelerometer(s) during waking hours (except when showering/bathing) for seven consecutive days, including weekdays and weekends. In the total DMD group, intensity cut-points for wrist-worn accelerometers were derived from a previous study and categorized as follows: sedentary 0–70; light PA 71–720; and MVPA ≥721 CPM.^19^ In ambulatory boys with DMD, the cut-points used in the Physical Activity in Norwegian Children Study (PANCS) were applied; this was a population study of physical activity levels in Norwegian children and adolescents,^20^ namely sedentary 0–99; light PA 100–1999; and MVPA ≥ 2000. Non-wear time was defined as ≥ 20 consecutive minutes with zero counts. After excluding data recorded from midnight to 6 a.m. (sleep time) and periods of non-wear time, a daily wear time of at least 480 min for a minimum of three days was considered valid for calculating participants’ overall PA.^21^ Participants were deemed compliant with the WHO's PA guidelines if they accumulated an average of at least 60 min of MVPA daily during the valid days of assessment.

### Secondary outcomes

Secondary outcome measures were assessed only in the intervention group during each DMD camp. Health-related QoL was measured using the Norwegian version of the Pediatric Quality of Life Inventory™ version 4.0 (PedsQL), a generic, age-specific self-reported questionnaire.^22^ These data were collected at baseline and at 12 months. Functional ability was assessed using the Performance of Upper Limb (PUL) v2.0 test at each DMD camp.^23^ Pulmonary function was measured with spirometry (e.g., forced vital capacity [FVC], slow vital capacity [SVC], forced expiratory volume in one second [FEV_1_] and peak cough flow [PCF]). Respiratory muscle strength was assessed through maximal inspiratory pressure (MIP) and maximal expiratory pressure (MEP). All pulmonary function tests were performed in upright, sitting, and supine positions. The best of three valid attempts was recorded.

The participants’ isometric hand-grip strength of both hands was measured using the K-Pull handheld dynamometer (Kinetec MedFac LTD, Aldershot, UK) following standardized procedures.^24^ Endurance was evaluated using a six-minute assisted bicycle test, with recordings of distance covered, peak heart rate, and perceived rating of exertion.^25^ All functional and physiological tests were conducted by trained and independent personnel.

### Statistics

Sample characteristics were quantified using descriptive statistics. Specifically, PA data, including wear time and time spent at different intensity levels, were reported as means with standard deviations for both the intervention group and the non-intervention group. PA outcomes were reported for the full cohort based on wrist-worn accelerometery, and separately for ambulatory participants based on waist-worn accelerometery. To evaluate within-group mean changes over time, paired samples t-tests were used. Between-group differences in change scores (expressed as absolute changes in daily minutes, consistent with public health recommendations such as the WHO guideline of ≥60 min daily MVPA) were analysed using an independent samples Welch's t-test allowing for unequal variances between groups. All statistical tests were two-sided, and p-values <0.05 were considered statistically significant. No adjustments were made for multiple comparisons. To complement p-values and account for limited statistical power due to small sample sizes, effect sizes were calculated using Cohen's d for paired and independent comparisons where applicable (small effect = 0.2, moderate effect = 0.5, large effect = 0.8). For wrist-worn accelerometer outcomes, Mann-Whitney U tests were additionally performed as sensitivity analyses for between-group comparisons. Due to the very small sample size in the ambulatory subgroup assessed with waist-worn accelerometers (n = 4/n = 5), these results were considered exploratory and are therefore presented descriptively only, without inferential or effect size estimation. All analyses were performed using IBM SPSS Statistics version 29 (Armonk, NY, USA). Visualizations of individual trajectories from baseline to follow-up in daily time spent in sedentary PA, light PA, and MVPA time among ambulatory participants in the intervention and non-intervention groups, based on waist-worn accelerometer data, were performed in R version 4.3.2. A mean trend line for each group (obtained from a simple linear regression model) was superimposed on the plot to illustrate overall patterns in PA over time.

## Results

**Figure 1.**
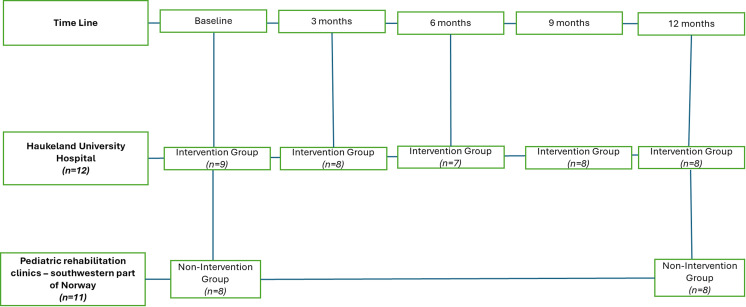
Accelerometer data obtained from the intervention and the non-intervention group at the different timepoints during follow-up.

Twenty-three boys with DMD volunteered to participate in the study, representing one-third of the Norwegian population of boys with DMD aged 6–18 years. Participants with valid PA-level data varied across timepoints during follow-up (Figure 1).

At baseline, one accelerometer malfunctioned, and two participants in the intervention group provided insufficient data. Across follow-up timepoints (three, six, nine, and 12 months), variation in valid data was due to insufficient numbers of valid wear days. In the non-intervention group (n = 11), valid accelerometer data were obtained from five ambulatory and three non-ambulatory participants. Data from three participants was missing due to logistical issues with device return. Among ambulatory boys in the intervention group, three participants experienced falls resulting in leg fractures, but not during the physical exercise training sessions. These injuries temporarily prevented valid hip-worn accelerometer recordings, but two boys regained ambulation and provided valid data at 12 months. The injuries contributed to reduced PA levels during follow-up. Eleven participants in the intervention group completed the study. One participant passed away during follow-up due to an acute illness unrelated to the intervention (Figure 1, Table 2).

### Baseline characteristics and PA levels

At baseline, differences in age and functioning (ambulatory versus non-ambulatory) were observed between the two groups (see Table 1). Both groups reported weekly physiotherapy, with stretching being an essential treatment focus. The non-intervention group showed higher participation in active transportation to school (e.g., walking or bicycling) and participation in physical education. The intervention group exhibited more variation in activity levels, with a notably higher percentage of participants reporting no PA in the past two weeks, especially in terms of hard activity (see Table 1).

**Table 1.** Baseline characteristics of the included participants with Duchenne muscular dystrophy.

Variables	Intervention group	Non-intervention group
**n**	n = 12	n = 11
**Age** (years)	14.2 ± 2.5	10.6 ± 3.8
**Height** (cm)	134.3 ± 14.2	127.3 ± 12.0
**Weight** (kg)	43.2 ± 13.8	41.2 ± 17.4
**Ambulatory boys** (n (%))	6 (50%)	8 (73%)
**Non-ambulatory boys** (n (%))	6 (50%)	3 (27%)
**Treatment**		
**Steroid treatment** (n (%))	10/12 (83%)	6/9 (55%)
**Physiotherapy (>1 time per week) (n (%))**	10/12 (80%)	7/9 (64%)
**Reported PA**		
**Active transport to school** (n (%))	1/12 (8%)	5/9 (55%)
**Physical education** (n (%))	2/12 (16%)	5/9 (55%)
**Swimming at school** (n (%))	3/12 (25%)	3/9 (33%)
**Leisure time PA last 12 months** (> 1 day per week and >20 min) (n (%))	6/12 (50%)	4/9 (44%)
Frequency of hard* or light** physical activity the last two weeks (>20 min)		
**No days** (n (%))	6/12 (50%) * - 1/12 (8%) **	6/9 (55%) * - 0 /9 (0%) **
**1–2 days** (n (%))	4/12 (33%) * - 3/12 (25%) **	2/9 (18%) * - 4/9 (44%%) **
**3–5 days** (n (%))	1/12 (8%) * - 4/12 (33%) **	1/9 (14%) * - 2/9 (22%) **
**6–8 days** (n (%))	1/12 (8%) * - 2/12 (16%) **	0/9 (18%) *- 2/9 (22%) **
**> 9 days** (n (%))	0/12 (8%) * - 2/12 (16%) **	0/9 (100%) * - 1/9 (12%) **

Data givens as mean ± standard deviation, n and percentages. cm: centimetres; kg: kilograms; PA: physical activity.

Table 2 presents accelerometer-measured PA levels over time during follow-up, while Table 3 shows the corresponding changes from baseline to 12 months.

**Table 2.** Accelerometer measured daily time (in minutes) spent in sedentary, light, and MVPA intensities during follow-up from baseline to 12 months.

PA levels (daily minutes)	Intervention group					Non-intervention group	
	Baseline	3 months	6 months	9 months	12 months	Baseline	12 months
**Wrist-worn accelerometer**							
Ambulatory and non-ambulatory participants (n)	n = 9/12	n = 8/12	n = 7/12	n = 8/11	n = 8/11	n = 8/8	n = 8/8
Wear time (minutes)	712.3 ± 81.2	749.63 ± 93.1	857.1 ± 141.1	809.3 ± 105.2	826.9 ± 99.1	817.2 ± 101.1	738.6 ± 48.1
Sedentary time (minutes)	502.2 ± 152.2	481.34 ± 167.9	592.7 ± 120.5	556.0 ± 157.1	565.9 ± 169.5	424.8 ± 117.5	428.8 ± 144.4
Light PA (minutes)	177.6 ± 91.1	218.36 ± 65.3	213.6 ± 91.0	206.5 ± 78.7	216.5 ± 89.2	273.3 ± 67.6	219.0 ± 77.7
MVPA (minutes)	32.6 ± 30.3	49.92 ± 35.0	50.7 ± 53.5	46.8 ± 42.7	44.5 ± 39.5	119.1 ± 83.3	90.8 ± 78.0
**Waist-worn accelerometer**							
Ambulatory participants (n)	n = 6 /6	n = 4/6	n = 3/6	n = 3/6	n = 4/6	n = 5/8	n = 5/8
Wear time (minutes)	702.6 ± 81.8	623.3 ± 95.2	790.1 ± 174.4	695.3 ± 147.9	692.8 ± 104.5	666.2 ± 62.2	673.9 ± 77.0
Sedentary time (minutes)	524.2 ± 81.4	505.4 ± 63.0	655.4 ± 141.7	585.0 ± 111.0	579.6 ± 58.1	449.2 ± 46.5	491.3 ± 44.3
Light PA (minutes)	136.7 ± 89.6	107.3 ± 51.5	123.8 ± 66.4	104.7 ± 62.4	104.3 ± 55.9	186.4 ± 79.3	164.6 ± 75.6
MVPA (minutes)	41.7 ± 69.4	10.6 ± 7.4	10.9 ± 9.8	5.7 ± 3.5	8.8 ± 6.0	30.5 ± 27.5	18.0 ± 10.2

Data includes both ambulatory and non-ambulatory participants (wrist-worn accelerometer data) and with changes in PA levels in the ambulatory boys of both groups, presented as daily minutes means ± SD.

PA: physical activity; MVPA; moderate-to-vigorous physical activity; SD: standard deviation.

**Table 3.** Accelerometer measured changes in daily PA levels from baseline to 12 months in the intervention and non-intervention groups.

PA levels (daily minutes)	Intervention group			Non-Intervention group				Between group differences		
	Change from baseline to 12 months	p-values (paired t-test)	Cohen`s d	Change from baseline to 12 months	p-values (paired t-test)	Cohen`s d	Mean difference (standard error)	p-values (Welch`s t-test)	Cohen`s d	p-values (Mann-Whitney U test)
**Wrist-worn accelerometer**										
Ambulatory and non-ambulatory participants (n)	n = 7/11	n = 7/11		n = 8/8	n = 8/8					
Wear time	90.2 ± 108.2	0.070		−78.5 ± 113.6	0.091		168.72 (57.3)	0.012		
Sedentary	25.0 ± 101.8	0.541	0.25	4.0 ± 157.6	0.954	0.25	20.94 (67.7)	0.762	0.16	0.563
Light	50.0 ± 117.6	0.304	0.43	−54.3 ± 55.5	0.028	−0.98	104.23 (48.6)	0.063	1.16	0.083
MVPA	15.3 ± 56.6	0.502	0.27	−28.3 ± 25.0	0.015	−1.13	43.54 (23.1)	0.096	1.02	0.105
**Waist-worn accelerometer**										
Ambulatory participants (n)	n = 4/11			n = 5/8						
Wear time	−37.0 ± 106.0			7.7 ± 16.4						
Sedentary	64.0 ± 103.2			42.1 ± 48.7						
Light	−51.0 ± 84.6			−21.8 ± 30.6						
MVPA	−50.2 ± 80.0			−12.6 ± 17.3						

Changes in daily time spent in sedentary time, light PA, and MVPA in both the intervention group and the non-intervention group from baseline to 12-month follow-up based on wrist-worn accelerometer data (both ambulatory and non-ambulatory participants) and waist-worn accelerometer data (ambulatory participants only). The within-group changes in PA levels from T0 to T4 (paired differences) are presented as means ± SD and tested using paired t-tests. Between-group change scores are presented as mean differences with standard errors and tested using independent samples Welch's t-tests. Effect sizes are reported as Cohen's d. Due to very small sample sizes (n = 4 and n = 5), waist-worn accelerometer results are presented descriptively as mean ± SD, and no inferential statistics or effect size estimates are reported. MVPA: moderate to vigorous physical activity; PA: physical activity, SD: standard deviation.

**Figure 2.**
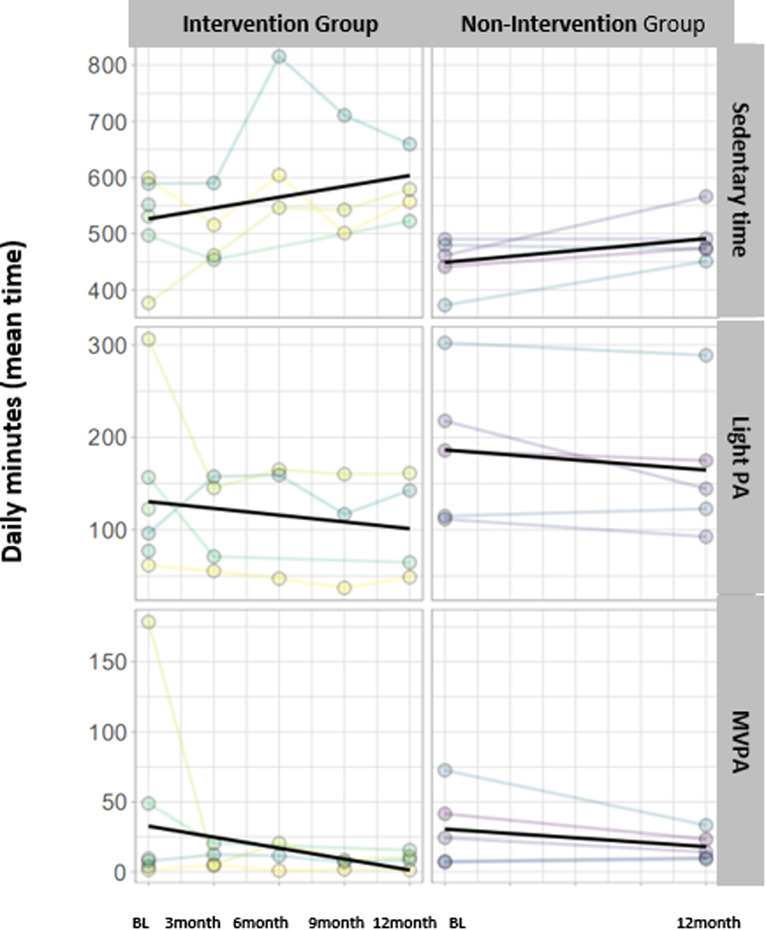
Individual trajectories and group-level trends (black line) in mean daily time spent in sedentary, light, and moderate-to-vigorous physical activity (MVPA) among ambulatory participants in the intervention and non-intervention groups, based on waist-worn accelerometer data. The y-axis indicates average daily time in minutes. BL = baseline.

Based on wrist-worn data from the full cohort, light PA, MVPA levels, and sedentary time increased slightly in the intervention group over the 12 months. The non-intervention group showed a reduction in both light PA and MVPA levels, while sedentary time remained stable. No between-group differences were observed (Table 3). Among ambulatory participants assessed with waist-worn accelerometers, both groups showed a general pattern of reduced time in light PA and MVPA levels and increased sedentary time over the 12 months (Table 2). Due to very small sample sizes (n = 4 and n = 5), these findings are presented descriptively (Table 3, Figure 2).

### Functional and patient-reported outcomes in the intervention group

The total PUL score remained relatively stable overall, though significant declines were noted at six and 12 months, primarily in the shoulder and elbow dimensions (Table 4). Pulmonary function, including FVC and SVC, showed a small decline from baseline to six months, with no further changes at 12 months. Measures of respiratory and muscle strength (MIP, MEP, and grip strength) fluctuated, indicating overall maintained functioning. Endurance, assessed via the assisted six-minute bicycle test, also remained stable throughout the study period. Health-related QoL (PedsQL total score) showed no significant change, though a small improvement was observed from baseline to 12 months. Overall, these findings suggest functional stability across multiple domains during the one-year exercise intervention (Table 4).

**Table 4.** Secondary outcome measures during follow-up in the intervention group. Data are presented as means ± SD and p-values referring to within-comparisons from baseline to 12 months (paired t-test).

Intervention group	Measurements						Within group difference					
	Baseline		6 months		12 months		Difference baseline – 6 months			Difference baseline – 12 months		
	*n*	*mean* *±* *SD*	*n*	*mean* *±* *SD*	*n*	*mean* *±* *SD*	*n*	*mean* *±* *SD*	*p-value*	*n*	*mean* *±* *SD*	*p-value*
**Performance of Upper Limb**												
D1 Shoulder	11	6.0 ± 5.8	11	5.6 ± 5.6	11	4.6 ± 4.9	11	−0.4 ± 0.7	0.104	11	−1.4 ± 2.2	0.072
D2 Elbow	11	12.8 ± 5.6	11	12.0 ± 6.1	11	11.6 ± 6.5	11	−0.8 ± 0.5	0.095	11	−1.3 ± 1.9	0.065
D3 Wrist	11	11.4 ± 2.4	11	10.8 ± 2.2	11	11.0 ± 2.4	11	−0.6 ± 1.0	0.095	11	−0.4 ± 0.8	0.167
Total score	11	30.3 ± 13.0	11	29.9 ± 13.3	11	27.5 ± 13.0	11	−0.4 ± 1.7	0.023	11	−2.8 ± 2.6	0.004
**Lung function**												
FVC (litres (%pred))	12	1.7 ± 0.6(73%)	12	1.6 ± 0.5(68%)	11	1.6 ± 0.6(67%)	12	−0.1 ± 0.4	0.019	11	−0.7 ± 0.2	0.209
FEV_1_(litres (%pred))	12	1.5 ± 0.1(70%)	12	1.5 ± 0.5(69%)	11	1.4 ± 0.5(64%)	12	−0.0 ± 0.2	0.990	11	−0.5 ± 0.2	0.387
SVC (litres)	12	1.7 ± 0.6	12	1.6 ± 0.5	11	1.6 ± 0.6	12	−0.1 ± 0.8	0.021	11	−0.0 ± 0.1	0.572
**Respiratory muscles**												
MIP (cm H_2_0)	9	45. ± 18.6	9	45.8 ± 18.1	9	45.1 ± 19.8	9	0.2 ± 9.4	0.945	9	−0.4 ± 9.0	0.886
MEP (cm H_2_0)	9	36.4 ± 17.0	9	40.2 ± 18.0	9	39.8 ± 21.7	9	3.8 ± 6.9	0.138	9	3.3 ± 11.9	0.428
CPF (litres/min)	11	241.9 ± 60.5	11	238.1 ± 54.2	10	244.7 ± 51.5	11	−3.8 ± 23.3	0.602	10	11.1 ± 17.9	0.083
**Isometric muscle strength**												
Grip strength right side (kg)	11	5.2 ± 2.2	11	5.1 ± 2.5	10	5.0 ± 2.6	11	−0.7 ± 0.9	0.797	10	0.1 ± 0.9	0.643
Grip strength left side (kg)	11	5.23 ± 2.5	11	5.0 ± 2.5	10	4.3 ± 2.1	11	−0.3 ± 0.5	0.110	10	−0.6 ± 0.9	0.070
**Six-minute assisted bicycle test**												
Distance (km)	11	2.1 ± 0.9	11	2.1 ± 1.0	11	1.8 ± 1.1	11	0.2 ± 0.2	0.724	11	−0.4 ± 0.8	0.182
OMNI score (0–10)	10	7.6 ± 3.4	10	7.1 ± 3.1	9	5.0 ± 3.9	10	−0.5 ± 2.3	0.513	9	−1.9 ± 3.7	0.160
HR_peak_	11	136.8 ± 50.6	11	142.6 ± 52.6	10	130.6 ± 67.5	11	5.6 ± 19.2	0.513	10	−6.2 ± 49.7	0.160
**PedsQL**												
Health and activities	11	17.4 ± 3.5		-	11	17.7 ± 4.3		-	-	11	0.4 ± 4.4	0.791
Feelings	11	6.1 ± 3.8		-	11	4.5 ± 3.1		-	-	11	−1.6 ± 2.8	0.085
Participation	11	5.8 ± 3.0		-	11	4.6 ± 2.7		-	-	11	−1.2 ± 3.3	0.268
Learning	11	6.8 ± 3.1		-	11	5.9 ± 2.3		-	-	11	−0.9 ± 2.4	0.242
PedsQL total score	11	36.1 ± 7.0		-	11	32.4 ± 8.6		-	-	11	−3.7 ± 10.5	0.265

D: dimension; FVC: forced vital capacity (absolute and %pred); FEV: forced expiratory volume after 1 s; SVC: slow vital capacity; MIP: maximal inspiratory pressure; MEP: maximal expiratory pressure; CPF: cough peak flow; kg: kilograms, PedsQL: Pediatric Quality of Life Inventory; km: kilometres; HR_peak_: maximal heart rate; SD: standard deviation; pred: predicted. “n” indicates the number of participants with valid observations for each outcome at each time point; n may vary due to missing data. “-“indicates outcomes not assessed at that time point (e.g., PedsQL at 6 months).

### Adverse events

No adverse events related to the home-exercise program were reported. Weekly training diary reports showed no sustained fatigue, occurrence of pain due to injuries or overload (VAS), or negative comments. Serum CK levels remained stable and within acceptable ranges at both the 2^nd^ and 3^rd^ DMD camps.

## Discussion

In this study, an individually tailored, home-based exercise program for boys with DMD was applied and proved to be both beneficial and safe. Based on wrist-worn accelerometery, an increase in both light PA and MVPA levels was observed over 12 months for the intervention group. The non-intervention group experienced a significant decline during the same period. Secondary outcomes indicated a decline in upper-limb function, but stable lung function, grip strength, and endurance capacity in the intervention group. QoL showed a small improvement in the intervention group, and no adverse events were attributed to the exercise program. Importantly, the intervention was designed to promote PA without accelerating disease progression, which is a central concern in DMD. These findings support this premise, as no safety signals or functional deterioration beyond expected disease-related changes were observed during the one-year intervention period.

### Impact on PA level and sedentary behaviour in DMD

Boys with DMD face unique barriers to PA, including progressive functional limitations, reduced access to adapted services, and misconceptions about exercise safety.^2^ These changes contribute to lower PA levels, elevated risk of obesity and cardiovascular disease, and reduced psychosocial well-being compared to their peers.^1^ Therefore, increasing PA levels in this group is important.

In the intervention group, a sustained increase in daily light PA and MVPA levels was observed, particularly during the first months of the program. Among the ambulatory boys, however, declines in light PA and MVPA were observed when measured by waist-worn accelerometers. Due to the very small sample size in the ambulatory subgroup assessed with waist-worn accelerometers (n = 4–5), these findings should be interpreted descriptively and considered exploratory. This was likely influenced by the adverse events not related to the training intervention, resulting in lower-limb fractures among three participants in the intervention group. These findings highlight important methodological differences, as discussed in previous studies.^7,26^ Waist-worn accelerometers primarily capture lower-limb movement and ambulatory activity, while wrist-worn accelerometers capture total body movement, including upper-limb activity, which remains relevant as ambulatory function declines.^7,26^ Most boys did not reach the WHO recommendation of 60 min of daily MVPA. However, even modest changes in PA levels may still provide meaningful health benefits.^1,2^ Importantly, increases in light and MVPA levels were maintained over 12 months, while the non-intervention group experienced a significant decline in both. Although several outcomes did not reach statistical significance, effect size estimates based on wrist-worn accelerometer data indicated a moderate effect on light PA. This suggests that even modest changes in activity patterns may be clinically meaningful in boys with DMD, given the progressive nature of the disease.

Despite varying functional challenges, all participants who completed the intervention adhered well to the program, indicating good exercise tolerance. The gradual progression in activity levels, combined with the use of autoregulatory tools (OMNI scale, RIR, Borg scale), likely supported individual adaptation and reduced risk of overload. Regular monitoring and close involvement of key support personnel enabled real-time PA adjustment and helped sustain motivation throughout the intervention period. Balancing training load and recovery is central in DMD due to the absence of dystrophin. Dystrophic muscles are characterized by increased fragility and susceptibility to injury, fibrosis, and contractures. Consequently, high-intensity and eccentric loading have generally been avoided in DMD.^9,27^ On the other hand, physical inactivity promotes disuse atrophy and deconditioning.^8,27,28^ Therefore, the exercise approach emphasized aerobic and dynamic movements, which formed the basis for the present intervention.

In this study, the observed increase in sedentary time alongside increases in light PA and MVPA levels may reflect a compensatory strategy to balance training demands and prevent fatigue. This is consistent with a broader understanding of fatigue as a multifactorial consequence of reduced aerobic capacity and ineffective muscle metabolism, rather than overuse alone.^29,30^

### Impact on function and health-related QoL

Lung function, grip strength, and endurance remained stable in the intervention group, while upper-limb function declined. A majority of the strength exercises involved a fixed flexed hand position, with limited activation of the extensors. Including tasks that promote wrist and finger extension may be beneficial, particularly to maintain activities of daily living and to counteract inactivity-related patterns such as prolonged sitting and screen time, as demonstrated in a recent study.^7^ The overall pattern of functional maintenance observed aligns with previous short-term studies (≤ 6 months), which reported early functional gains in previously inactive individuals, likely reflecting neuromuscular adaptation during the first 2–3 months of training.^27,28,31,32^ Recent short-term trials have also demonstrated modest improvements following aerobic, trunk-focused, or cycling interventions.^33–36^ To the authors’ knowledge, only one previous study has assessed the long-term effects of exercise training in this population.^37^ Consistent with those findings, the current study's results suggest that, while functional gains may occur, continued improvements are uncommon beyond this initial phase, with functional stabilization representing a more realistic long-term outcome. Several studies suggest that regular PA may slow functional decline compared to non-exercising controls even without measurable functional gains.^27,28^ Heutinck et al. found no significant change in total performance of upper limb scores but indicated delayed loss of strength and range of motion,^28^ while Jansen et al. suggested that assisted cycling may counteract disuse effects despite ongoing disease progression in DMD populations. These findings are consistent with the observations in this study. Stabilization of cardiopulmonary parameters and overall PA levels may indicate a protective role of structured and individualized exercise training.

This is further supported by Lott et al., who demonstrated that boys with higher habitual step activity were significantly more likely to retain ambulation two years later, suggesting that maintaining higher daily PA levels may have long-term protective effects on ambulation and overall functional preservation in DMD populations.^38^ Even though no significant changes were observed in PedsQL, improvements in health-related QoL were reported by the participants, consistent with Heutinck et al., who also observed higher QoL scores in their intervention group during follow-up.^12^ These findings underscore that psychosocial benefits of PA, such as increased well-being, may be equally important as physical outcomes, and that interventions should be studied with a broad methodological approach.^39^

### Suitability and safety

The one-year exercise intervention, with two weekly strength sessions and one weekly endurance session, was suitable for most DMD participants. Progressive loading up to moderate intensity was well-tolerated. Although resource-intensive, key elements such as weekly follow-up, tailored prescriptions, structured supervision, and strong involvement by key support personnel contributed to high adherence and real-world applicability in this study. These findings are consistent with previous studies, where frequent monitoring, adaptive feedback, and active caregiver involvement supported program feasibility in short-term interventions.^27,28,31^ Further, previous studies in DMD and neuromuscular rehabilitation emphasize that social interaction, peer support, and individualized supervision can enhance motivation, adherence, and well-being during both short and long-term training interventions.^4,27,28,39^ Although progressive loading was the overall goal of the strength training program, temporary regressive adjustments were occasionally required to accommodate individual fluctuations in functional capacity and/or motivation. For non-ambulatory participants, adaptation commonly included modified positioning and additional support to enable safe execution of the exercises. These adjustments were guided by the use of autoregulatory tools (OMNI scale and RIR) and weekly monitoring of training diaries. It could be speculated that strength loss and the need to reduce loading are the best measures of muscle injury. If there was no need to reduce loads, this could be considered an example of resistance exercise being safe for boys with DMD.

CK levels remained within acceptable limits throughout the intervention.

A central concern regarding exercise in DMD is the potential risk of accelerating disease progression due to increased muscle fragility; these safety findings indicate that the exercise program did not accelerate disease progression. This interpretation is further supported by overall maintenance of pulmonary function, muscle strength, and endurance capacity during the one-year intervention period, while the observed decline in upper-limb function was consistent with the expected natural disease course.

Three participants experienced accidental falls resulting in lower-limb fractures that were unrelated to the intervention itself but are common in this population due to osteoporosis, muscle weakness and contractures.^40^ Following individualized adjustments, these participants continued their programs with modified exercises, and two regained ambulation. Based on these observations, the intervention was considered safe.

### Implication for future studies and practice

Finding the optimal balance between exercise loading and recovery remains central. Both overprotection and excessive loading may be detrimental in DMD. As supported by previous studies,^8,27,28^ many boys experience fatigue at relatively low workloads due to deconditioning, not muscle injury per se. Appropriately dosed moderate-intensity exercise may improve tolerance over time. Future studies should investigate whether slightly higher training frequencies, earlier intensity progression (e.g., OMNI 5–6), or increasing non-exercise daily PA levels may further support function. Continued use of autoregulatory tools (OMNI, RIR, Borg scale) remains crucial for safe, personalized exercise dosing.

A complementary project conducted after completion of the intervention explored participant and key personnel experiences and has been published elsewhere.^39^ Incorporating the user perspective may further inform the development and implementation of sustainable, home-based exercise interventions in DMD. To improve scalability and affordability, future studies should also explore less resource-intensive follow-up models, including digital support solutions, while maintaining safety through individualized dosing and autoregulatory tools. Additionally, addressing recovery, nutrition, sleep, and behavioural motivation will be essential for optimizing adherence and long-term outcomes.

### Study limitations

This study has several limitations. As the study was not randomized, baseline differences between the intervention and non-intervention groups may have influenced the observed outcomes and limited causal interpretation. The small sample size, including both ambulatory and non-ambulatory participants, introduced heterogeneity and limited statistical power with an increased risk of type II errors,^41^ and contributed to considerable individual variability in PA levels within both groups. Ideally, the use of age and function matched controls or a cross-over design would have improved internal validity.^42^ Moreover, the possibility that participants in the non-intervention group were not fully representative of the wider DMD population cannot be excluded, as some may have been particularly motivated, potentially introducing selection bias. Further, the non-intervention group was only assessed at baseline and 12 months. More frequent follow-up of both groups could have allowed better comparisons over time, but this was not possible within the available resources and logistical constraints. Although assessors were not involved in delivering the intervention, lack of blinding may have introduced detection bias.^43^ Finally, natural variability in disease progression likely contributed to the diverse responses observed across participants.

In addition, the subgroup of ambulatory participants assessed with waist-worn accelerometers was small (intervention group n = 4; non-intervention group n = 5), which limited statistical power and precluded meaningful between-group inference. Accordingly, waist-worn accelerometer results are presented descriptively, interpreted as exploratory, and considered secondary to the wrist-worn accelerometer findings derived from the full cohort.

The present study provides valuable insight into the suitability, safety, and potential benefits of a long-term, home-based exercise program in boys with DMD. However, taken together, the limitations indicate that it was not designed to provide definitive evidence regarding the efficacy of structured exercise training on disease progression or long-term functional outcomes. Accordingly, causal inference should be made with caution, and larger, adequately powered studies are needed to determine the extent to which programmed exercise may influence clinical outcomes in this population.

## Conclusions

A one-year home-based exercise program including both strength and endurance training at light to moderate intensity appears beneficial, safe, and sustainable for boys with DMD. The intervention was associated with increased daily light PA and MVPA, stable function, and improved health-related QoL. The findings support the role of structured, individualized exercise as part of long-term clinical care in DMD, to promote PA and functional maintenance in this population.

## Supplemental Material

sj-docx-1-prm-10.1177_18758894261446029 - Supplemental material for Home-based exercise training in boys with Duchenne muscular dystrophy: A 12-month intervention study Supplemental material, sj-docx-1-prm-10.1177_18758894261446029 for Home-based exercise training in boys with Duchenne muscular dystrophy: A 12-month intervention study by Stian Hammer, Maria Vollsæter, Michel Toussaint, Ola Drange Røksund, Guri Brekke, Bjørge Herman Hansen, Haakon Krisitian Kvidaland, Lars Peder Bovim, Roy Miodini Nilsen and Tiina Andersen in Journal of Pediatric Rehabilitation Medicine
