# Supplementary material for: Home-based exercise training in boys with Duchenne muscular dystrophy: A 12-month intervention study
Source: J Pediatr Rehabil Med. 2026 May 11;19(3):217–30. doi: 10.1177/18758894261446029 (PMC13442673; doi:10.1177/18758894261446029)
Supplement: Supplementary file 1 [file sj-docx-1-prm-10.1177_18758894261446029.docx]

1. [**Push chair with load**](https://exorlive.com/video/?culture=en-US&ex=1203791)

Grasp handels on the chair. Maintain the grip and bend forwards with bent arms. Assume a stable body position. Take a deep breath, activate your abdominal muscles and push forward.

Remember to breathe throughout the exercise.

**Load: 12,5 kilo , Repetitions (steps): 16 , Sets: 2 , Pause: 3 minutes , OMNI scale: 4 , RiR: 6 , VAS scale: 0**

2. [**Sideways steps with miniband**](https://exorlive.com/video/?culture=en-US&ex=1203793)

Stand upright on the floor with feet positioned at hip width and an elastic band around your ankles. Walk sideways, preferably with your feet rotated inwards. Keep tension in the band during the exercise.

**Load: elasctic miniband , Repetitions (steps): 10 , Sets: 2 (right side and left side) , Pause : 0 , OMNI scale: 4 , RiR: 6 , VAS scale : 0**

3. [**Seated pulldown with elastic band**](https://exorlive.com/video/?culture=en-US&ex=1203811)

Sit on a chair with an elastic band attached above you. Hold the elastic band with both hands above your head, keep your back straight and pull the elastic band down. Keep your shoulder blades together throughout the exercise. Return and repeat.

Breathe in on top and out when back on top. Repeat. **Load: Elastic band , Repetitions: 10 , Sets: 1 , Pause: 3 minutes , OMNI scale: 4 , RiR: 6 , VAS Scale: 0**

4. [**Seated low row with elastic bands**](https://exorlive.com/video/?culture=en-US&ex=1204985)

Seated in chair in vertical position with elestic band tight around the upper back. Breathe in. Active abdominal muscles. Breath out when back in start position. Press your upper body back slowly return to the start position.

**Load: Elastic band , Repetitions: 12 , Sets: 1 , Pause: 0 , OMNI scale: 4 , RiR: 6 , VAS scale: 0**

5. [**Seated one arm right and left chest press with elastic**](https://exorlive.com/video/?culture=en-US&ex=1204672) [**band**](https://exorlive.com/video/?culture=en-US&ex=1204672)

Place an elastic band around the backrest of a chair. Sit on the edge of the chair with one end of the band in each hand. Begin with your arms elevated laterally and your elbows 90° flexed. Your fists are pointing forward. From this position, extend your elbows and move your arms straight forward at chest level. Slowly return to starting position.

**Load: Eleastic band , Repetitions: 11 , Sets: 1 , Pause: 3 minutes , OMNI scale: 4 , RiR: 6 , VAS scale: 0**

6. [**Seated back extensions**](https://exorlive.com/video/?culture=en-US&ex=1203816)

# Seated in chair in vertical position with elestic band tight around the upper back. Breathe in. Active abdominal muscles. Breath out when back in start position. Press your upper body back slowly return to the start position.

**Load: Elastic band , Repetitions : 15 , Sets: 1 , Pause: 0 , OMNI scale: 4 , RiR: 6 , VAS scale: 0**

Changes in program from baseline:

- added sit to stand
- removed walk with minibands

**minutes , OMNI scale: 5-6 , RiR: 5-4 , VAS scale: 0**

2. [**Sit to stand**](https://exorlive.com/video/?culture=en-US&ex=1204671)

Sit in the chair. Keep your feet on the floor. Raise up form the chair and sit down. Repeat.

**Load: Body weight , Repetitions: 4 , Sets: 2 , Pause: 3 , OMNI scale: 5-6 , RiR: 5-4 , VAS scale: 0**

3. [**Seated back extensions**](https://exorlive.com/video/?culture=en-US&ex=1203816)

# Seated in chair in vertical position with elestic band tight around the upper back. Breathe in. Active abdominal muscles. Breath out when back in start position. Press your upper body back slowly return to the start position.

**Load: Elastic band , Repetitions : 16 , Sets: 2 , Pause: 2 , OMNI scale: 5-6 , RiR: 5-4 , VAS scale: 0**

4. [**Seated low row with elastic bands**](https://exorlive.com/video/?culture=en-US&ex=1204985)

Seated in chair in vertical position with elestic band tight around the upper back. Breathe in. Active abdominal muscles. Breath out when back in start position. Press your upper body back slowly return to the start position.

**Load: Elastic band , Repetitions: 8 , Sets: 2 , Pause: 0 , OMNI scale: 5-6 , RiR: 5-4 , VAS scale: 0**

5. [**Seated pulldown with elastic band**](https://exorlive.com/video/?culture=en-US&ex=1203811)

Sit on a chair with an elastic band attached above you. Hold the elastic band with both hands above your head, keep your back straight and pull the elastic band down. Keep your shoulder blades together throughout the exercise. Return and repeat.

Breathe in on top and out when back on top. Repeat. **Load: Elastic band , Repetitions: 8 , Sets: 2 , Pause: 2 minutes , OMNI scale: 5-6 , RiR: 5-4 , VAS Scale: 0**

6. [**Seated one arm right and left chest press with elastic**](https://exorlive.com/video/?culture=en-US&ex=1204672) [**band**](https://exorlive.com/video/?culture=en-US&ex=1204672)

Place an elastic band around the backrest of a chair. Sit on the edge of the chair with one end of the band in each hand. Begin with your arms elevated laterally and your elbows 90° flexed. Your fists are pointing forward. From this position, extend your elbows and move your arms straight forward at chest level. Slowly return to starting position.

**Load: Eleastic band , Repetitions: 10 , Sets: 2 , Pause: 2 minutes , OMNI scale: 5-6 , RiR: 5-4 , VAS scale: 0**

7. [**Seated sit ups with elastic band**](https://exorlive.com/video/?culture=en-US&ex=1203817)

Place the elastic band around the ribcage. Breath in and active abdominal muscle. Keep a proud posture while bending forward. Breath out when in start postitions again. Reload air and repeat movement.

**Load: Elastic band , Repetitions : 6 , Sets: 2 , Pause: 20 sek , OMNI scale: 5-6 , RiR: 5-4 , VAS Scale: 0**

Changes in program from 6 month program

- added hammercurl and triceps curl

**minutes , OMNI scale: 5-6 , RiR: 5-4 , VAS scale: 0**

2. [**Sit to stand**](https://exorlive.com/video/?culture=en-US&ex=1204671)

Sit in the chair. Raise and sit down.

**Load: Body weight , Repetitions: 4 , Sets: 2 , Pause: 3 , OMNI scale: 5-6 , RiR: 5-4 , VAS scale: 0**

3. [**Seated back extensions**](https://exorlive.com/video/?culture=en-US&ex=1203816)

# Seated in chair in vertical position with elestic band tight around the upper back. Breathe in. Active abdominal muscles. Breath out when back in start position. Press your upper body back slowly return to the start position.

**Load: Elastic band , Repetitions : 16 , Sets: 2 , Pause: 2 minutes , OMNI scale: 5-6 , RiR: 5-4 , VAS scale: 0**

4. [**Seated low row with elastic bands**](https://exorlive.com/video/?culture=en-US&ex=1204985)

Seated in chair in vertical position with elestic band tight around the upper back. Breathe in. Active abdominal muscles. Breath out when back in start position. Press your upper body back slowly return to the start position.

**Load: Elastic band , Repetitions: 8 , Sets: 2 , Pause: 2 , OMNI scale: 5-6 , RiR: 5-4 , VAS scale: 0**

5. [**Seated pulldown with elastic band**](https://exorlive.com/video/?culture=en-US&ex=1203811)

Sit on a chair with an elastic band attached above you. Hold the elastic band with both hands above your head, keep your back straight and pull the elastic band down. Keep your shoulder blades together throughout the exercise. Return and repeat.

Breathe in on top and out when back on top. Repeat. **Load: Elastic band , Repetitions: 8 , Sets: 2 , Pause: 2 minutes , OMNI scale: 5-6 , RiR: 5-4 , VAS Scale: 0**

6. [**Seated one arm right and left chest press with elastic**](https://exorlive.com/video/?culture=en-US&ex=1204672) [**band**](https://exorlive.com/video/?culture=en-US&ex=1204672)

Place an elastic band around the backrest of a chair. Sit on the edge of the chair with one end of the band in each hand. Begin with your arms elevated laterally and your elbows 90° flexed. Your fists are pointing forward. From this position, extend your elbows and move your arms straight forward at chest level. Slowly return to starting position.

**Load: Eleastic band , Repetitions: 10 , Sets: 2 , Pause: 2 minutes , OMNI scale: 5-6 , RiR: 5-4 , VAS scale: 0**

7. [**Seated alternate hammercurl with cuff in superset**](https://exorlive.com/video/?culture=en-US&ex=1204164)

Let your arms hang down to your sides and in a neutral position. Start the movement by bending at the elbow and lifting the hand up before controlling the movement down to hanging again.

Right arm hammercurl and then ticeps kick back. Then left arm hammercurl and triceps kick back. No pause. Breath continually. **Load: 0,5 kilos , Repetitions: 8/4 , Sets: 1 , Pause: 0 , OMNI scale: 5-6 , RiR: 5-4 , VAS scale: 0**

8. [**Seated triceps kick with cuff in superset**](https://exorlive.com/video/?culture=en-US&ex=1204669)

Stand supported on one arm and one leg. Place the active arm beside your upper body, elbow bent 90 degrees and dumbbell hanging straight down. Now straighten your arm fully at your elbow. Slowly return to the start position and repeat.

**Load: 0,5 kilos , Repetitions: 8 , Sets: 1 , Pause: 0 , OMNI scale : 4-6 , RiR: 6-4 , VAS scale: 0**

9. [**Seated sit ups with elastic band**](https://exorlive.com/video/?culture=en-US&ex=1203817)

Place the elastic band around the ribcage. Breath in and active abdominal muscle. Keep a proud posture while bending forward. Breath out when in start postitions again. Reload air and repeat movement.

**Load: Elastic band , Repetitions : 6 , Sets: 2 , Pause: 20 sek , OMNI scale: 5-6 , RiR: 5-4 , VAS Scale: 0**

Exercise in wheelchair

1. [**Knee extension with elastic band**](https://exorlive.com/video/?culture=nb-NO&ex=1205973)

Sit with a band around your ankle, attached to an anchor point behind you. Sit with your back to the anchor point. Extend your knee without lifting your thigh from the chair seat. Return your leg to the starting position in a controlled manner. Repeat.

**Load: Elastic band , Repetitions: 8/10 , Sets: 1 , Pause: 0 , OMNI scale: 4 , RiR: 4 , VAS scale: 0**

2. [**Low row with elastic bands**](https://exorlive.com/video/?culture=nb-NO&ex=1204985)

Seated in vertical position with elestic band tight around the upper back. Breathe in. Active abdominal muscles. Breath out when back in start position. Press your upper body back slowly return to the start position.

**Load: Elastic band , Repetitions: 9/8 , Sets: 1 , Pause: 0 , OMNI scale: 4 , RiR: 4 , VAS scale: 0**

3. [**Chest press with elastic band**](https://exorlive.com/video/?culture=nb-NO&ex=1204672)

Place an elastic band around the backrest of a chair. Sit on the edge of the chair with one end of the band in each hand. Begin with your arms elevated laterally and your elbows 90° flexed. Your fists are pointing forward. From this position, extend your elbows and move your arms straight forward at chest level. Slowly return to starting position.

**Load: Eleastic band , Repetitions: 11 , Sets: 1 , Pause: 3 minutes , OMNI scale: 4 , RiR: 4 , VAS scale: 0**

4. [**Side bend with elastic band**](https://exorlive.com/video/?culture=nb-NO&ex=1205052)

**Load: Elastic band , Repetitions : 8/8 , Sets: 1 , Pause: 0 , OMNI scale: 4 , RiR: 4 , VAS scale: 0**

5. [**Back extensions**](https://exorlive.com/video/?culture=nb-NO&ex=1203816)

# Seated in chair in vertical position with elestic band tight around the upper back. Breathe in. Active abdominal muscles. Breath out when back in start position. Press your upper body back slowly return to the start position.

**Load: Elastic band , Repetitions : 10 , Sets: 2 , Pause: 2 , OMNI scale: 4 , RiR: 4 , VAS scale: 0**

6. [**Sit ups with elastic band**](https://exorlive.com/video/?culture=nb-NO&ex=1203817)

Place the elastic band around an ancor point and over your chest. Breath in, active abdominal muscles while you push the body foward. Return slowly and controled to the start positions. Breath out. Repeat.

**Load: Elastic band , Repetitions : 10 , Sets: 2 , Pause: 1 , OMNI scale: 4 , RiR: 4 , VAS Scale: 0**

7. [**Hip adduction in sling**](https://exorlive.com/video/?culture=nb-NO&ex=1206455)

Lie on the side with a sling around the calf of the upper leg. Put the hands under the head for support. Stabilise the stomach and lower back. Lift the hips by pushing the upper leg down in sling. At the same time lift the lower leg up against the other. Slowly return to the starting position.

**Load: Body weight , Repetitions: 5/5 , Sets: 1 , Pause: 0 , OMNI scale: 4 , RiR: 4 , VAS scale: 0**

8. [**Hip extention**](https://exorlive.com/video/?culture=nb-NO&ex=1206458)

Lie on the side with a sling placed around one foot. Moved the foot foward and then do a hip extention. Repeat.

**Load: Body weight , Repetitons: 6/6 , Sets: 1 , Pause: 0 , OMNI scale: 4 , RiR: 4 , VAS scale: 0**

Exercise in wheelchair

1. [**Knee extension with elastic band**](https://exorlive.com/video/?culture=nb-NO&ex=1205973)

Sit with a band around your ankle, attached to an anchor point behind you. Sit with your back to the anchor point. Extend your knee without lifting your thigh from the chair seat. Return your leg to the starting position in a controlled manner. Repeat.

**Load: Elastic band , Repetitions: 8/10 , Sets: 1 , Pause: 0 , OMNI scale: 5-6 , RiR: 2 , VAS scale: 0**

2. [**Low row with elastic bands**](https://exorlive.com/video/?culture=nb-NO&ex=1204985)

Breathe in. Active abdominal muscles. Breath out when back in start position. Press your upper body back slowly return to the start position.

**Load: Elastic band , Repetitions: 9/8 , Sets: 1 , Pause: 0 , OMNI scale: 5-6 , RiR: 2 , VAS scale: 0**

3. [**Chest press with elastic band**](https://exorlive.com/video/?culture=nb-NO&ex=1204672)

Place an elastic band around the backrest of a chair. Sit on the edge of the chair with one end of the band in each hand. Begin with your arms elevated laterally and your elbows 90° flexed. Your fists are pointing forward. From this position, extend your elbows and move your arms straight forward at chest level. Slowly return to starting position.

**Load: Eleastic band , Repetitions: 7/8 , Sets: 1 , Pause: 0 , OMNI scale: 5-6 , RiR: 2 , VAS scale: 0**

4. [**Side bend with elastic band**](https://exorlive.com/video/?culture=nb-NO&ex=1205052)

Stand with one foot approx. in the middle of the elastic band, hold the handle in your hand of the same side. Lower your upper body to that side and pull to opposite side. Change sides.

**Load: Elastic band , Repetitions : 8/8 , Sets: 1 , Pause: 0 , OMNI scale: 5-6 , RiR: 2 , VAS scale: 0**

5. [**Back extensions**](https://exorlive.com/video/?culture=nb-NO&ex=1203816)

# Seated in chair in vertical position with elestic band tight around the upper back. Breathe in. Active abdominal muscles. Breath out when back in start position. Press your upper body back slowly return to the start position.

**Load: Elastic band , Repetitions : 10 , Sets: 1 , Pause: 0 , OMNI scale: 5-6 , RiR: 2 , VAS scale: 0**

6. [**Sit ups with elastic band**](https://exorlive.com/video/?culture=nb-NO&ex=1203817)

Place the elastic band around an ancor point and over your chest. Breath in, active abdominal muscles while you push the body foward. Return slowly and controled to the start positions. Breath out. Repeat.

**Load: Elastic band , Repetitions : 8 , Sets: 1 , Pause: 0 , OMNI scale: 5-6 , RiR: 2 , VAS Scale: 0**

7. [**Hip adduction in a sling**](https://exorlive.com/video/?culture=nb-NO&ex=1206455)

Lie on the side with a sling around the calf of the upper leg. Put the hands under the head for support. Stabilise the stomach and lower back. Lift the hips by pushing the upper leg down in sling. At the same time lift the lower leg up against the other. Slowly return to the starting position.

**Load: Body weight , Repetitions: 5/5 , Sets: 1 , Pause: 0 , OMNI scale: 5-6 , RiR: 2 , VAS scale: 0**

8. [**Hip extension in sling**](https://exorlive.com/video/?culture=nb-NO&ex=1206458)

Lie on the side with a sling placed around one foot.Active abdominal muscels. Breath in. Moved the foot foward and then do a hip extention. Breath out. Repeat.

**Load: Body weight , Repetitions: 6/6 , Sets: 1 , Pause: 0 , OMNI scale: 5-6 , RiR: 2 , VAS scale: 0**

Exercise in wheelchair

1. [**Knee extension with elastic band**](https://exorlive.com/video/?culture=nb-NO&ex=1205973)

Attached the band to an anchor point behind you and place the band around one ankle. Breath in. Extend your knee without lifting your thigh from the chair seat. Return your leg to the starting position in a controlled manner while breathing out. Repeat.

**Load: Elastic band , Repetitions: 7/12 , Sets: 1 , Pause: 0 , OMNI scale: 5-6 , RiR: 3-2 , VAS scale: 0**

2. [**Low row with elastic bands**](https://exorlive.com/video/?culture=nb-NO&ex=1204985)

Attache the elastic band to an anchor point. Hold the elastic band in your hands. Breathe in. Active abdominal muscles. Pull chest forward and arm backward in a vertical row. Breath out when back in start position.Repeat

**Load: Elastic band , Repetitions: 11/10 , Sets: 1 , Pause: 0 , OMNI scale: 5-6 , RiR: 3-2 , VAS scale: 0**

3. [**Chest press with elastic band**](https://exorlive.com/video/?culture=nb-NO&ex=1204672)

Place an elastic band around the backrest of a chair. Sit on the edge of the chair with one end of the band in each hand. Begin with your arms elevated laterally and your elbows 90° flexed. Your fists are pointing forward. From this position, extend your elbows and move your arms straight forward at chest level. Slowly return to starting position.

**Load: Eleastic band , Repetitions: 10/10 , Sets: 1 , Pause: 0 , OMNI scale: 5-6 , RiR: 3-2 , VAS scale: 0**

4. [**Side bend with elastic band**](https://exorlive.com/video/?culture=nb-NO&ex=1205052)

Attache the elastic band to an anchor point. Hold the elastic band in your hand. Active abdominal muscles Breath in. Lower your upper body to that side and pull to opposite side. Return til start posititions and breath out. Repeat.

**Load: Elastic band , Repetitions : 13/13 , Sets: 1 , Pause: 0 , OMNI scale: 5-6 , RiR: 3-2 , VAS scale: 0**

5. [**Back extensions**](https://exorlive.com/video/?culture=nb-NO&ex=1203816)

Sit in vertical position with elestic band tight around the upper back. Breathe in. Active abdominal muscles. Press your upper body back slowly return to the start position. Breath out when back in start position.Repeat.

**Load: Elastic band , Repetitions : 12 , Sets: 1 , Pause: 0 , OMNI scale: 5-6 , RiR: 3-2 , VAS scale: 0**

6. [**Sit ups with elastic band**](https://exorlive.com/video/?culture=nb-NO&ex=1203817)

Place the elastic band around an ancor point and over your chest. Breath in, active abdominal muscles while you push the body foward. Return slowly and controled to the start positions. Breath out. Repeat.

**Load: Elastic band , Repetitions : 12 , Sets: 1 , Pause: 0 , OMNI scale: 5-6 , RiR: 3-2 , VAS Scale: 0**

**Supplementary 4**

**Examples of Exercise Equipment used by some of the participants in their strength training sessions**.

THERABAND (Akron, OH, USA)	(Ballerup, Denmark)	Exertubes with Handles, (Klubben,

Norway)

Weighed cuffs (wrist or ancle)	Hand weights Biltema Nordic Services AB (Halmstad, Sweden)

**Supplementary 4**

**Examples of Exercise Equipment used by some of the participants in their endurance training.**

THERA-Trainer Tigo 510 with 10,4 inches screen (Medica Medizintechnik GmbH, Hockdorf, Germany)

MOTOmed (RECK-Tecknik GmbH & Co, Betzenweiler, Germany)

Innowalk, (Made for Movement, Skien, Norway)
